# Transcriptomic Analysis of Insulin-Sensitive Tissues from Anti-Diabetic Drug Treated ZDF Rats, a T2DM Animal Model

**DOI:** 10.1371/journal.pone.0069624

**Published:** 2013-07-26

**Authors:** Yo Na Kim, Sangok Kim, Il-Yong Kim, Jae Hoon Shin, Sooyoung Cho, Sun Shin Yi, Wan Kyu Kim, Kyung-Sub Kim, Sanghyuk Lee, Je Kyung Seong

**Affiliations:** 1 Laboratory of Developmental Biology and Genomics, College of Veterinary Medicine, Research Institute for Veterinary Science, BK21 Program for Veterinary Science, Seoul National University, Seoul, Korea; 2 Interdisciplinary Program for Bioinformatics, Program for Cancer Biology and BIO-MAX Institute, Seoul National University, Seoul, Korea; 3 Department of Biomedical Laboratory Science, College of Medical Sciences, Soonchunhyang University, Asan, Chungnam, Korea; 4 Ewha Research Center for Systems Biology, Division of Molecular and Life Sciences, Ewha Womans University, Seoul, Korea; 5 Department of Biochemistry and Molecular Biology, Integrated Genomic Research Center for Metabolic Regulation, Institute of Genetic Science, Brain Korea 21 Project for Medical Science, Yonsei University College of Medicine, Seoul, Korea; University of KwaZulu-Natal, South Africa

## Abstract

Gene expression changes have been associated with type 2 diabetes mellitus (T2DM); however, the alterations are not fully understood. We investigated the effects of anti-diabetic drugs on gene expression in Zucker diabetic fatty (ZDF) rats using oligonucleotide microarray technology to identify gene expression changes occurring in T2DM. Global gene expression in the pancreas, adipose tissue, skeletal muscle, and liver was profiled from Zucker lean control (ZLC) and anti-diabetic drug treated ZDF rats compared with those in ZDF rats. We showed that anti-diabetic drugs regulate the expression of a large number of genes. We provided a more integrated view of the diabetic changes by examining the gene expression networks. The resulting sub-networks allowed us to identify several biological processes that were significantly enriched by the anti-diabetic drug treatment, including oxidative phosphorylation (OXPHOS), systemic lupus erythematous, and the chemokine signaling pathway. Among them, we found that white adipose tissue from ZDF rats showed decreased expression of a set of OXPHOS genes that were normalized by rosiglitazone treatment accompanied by rescued blood glucose levels. In conclusion, we suggest that alterations in OXPHOS gene expression in white adipose tissue may play a role in the pathogenesis and drug mediated recovery of T2DM through a comprehensive gene expression network study after multi-drug treatment of ZDF rats.

## Introduction

Type 2 diabetes mellitus (T2DM) is a metabolic disorder that is primarily characterized by insulin resistance and hyperglycemia [1]. T2DM affects over 110 million people worldwide and is a principal contributor to diabetic vascular disease, including atherosclerosis and diabetic retinopathy, which causes blindness [2]. A main component of T2DM is insulin resistance. Insulin does not control glucose utilization in fat and muscle cells in patients with T2DM, which causes hyperglycemia [3]. Thus, the pancreas produces more insulin and the cells become even more resistant, resulting in high glucose levels with hyperinsulinemia in patients with T2DM. However, the molecular pathogenesis causing insulin resistance is not fully understood. Therefore, searching for differentially expressed genes involved in the onset of insulin resistance in insulin-sensitive and insulin-producing tissues from patients with diabetes may help us to understand the molecular mechanisms involved in T2DM.

Several anti-diabetic therapeutics have been developed in accordance with the targeting molecules driving T2DM. Metformin is a widely used anti-diabetic and hyperglycemic agent that reduces hepatic glucose synthesis and improves insulin sensitivity in peripheral tissues [4]. In addition, metformin improves metabolic variables such as dyslipidemia and fibrinolysis [5]. Rosiglitazone, another anti-diabetic agent, is an artificial ligand of peroxisome proliferator-activated receptor gamma that improves insulin sensitivity [6]–[9] and mitochondrial function [10], [11] in peripheral tissues. Glimepiride, the latest second-generation sulfonylurea for treating T2DM, increases pancreatic β-cell function to stimulate insulin secretion resulting in a hypoglycemic action [12]. However, until now, few studies have used cDNA microarray to compare expression profiles between patients using different kinds of anti-diabetic drugs.

Expression profiling by DNA microarray allows a survey of genome-wide transcriptomic changes and determines which changes are expressed in particular disease states such as cancer [13]. Microarray data can be used to classify individuals according to molecular characteristics and to generate hypotheses about disease mechanisms. Studies using microarray have been performed on muscle [14]–[16], liver [4], and adipose tissue to identify candidate genes related to diabetes [17]. Differentially regulated transcripts have been determined in anti-diabetic drug-treated diabetic animal models using DNA microarray to validate the functions of the discovered genes [7], [18].

An animal model of human disease is a useful tool to study the pathogenesis of human disease. The Zucker diabetic fatty (ZDF) rat is a well-characterized obesity and T2DM-animal model with a point mutation in the leptin receptor. The homozygote recessive male rat becomes obese and hyperinsulinemic at 7 weeks of age. As the rats grow older, they show diabetic phenotypes accompanied by hyperglycemia and develop diabetes at 12 weeks of age. Therefore, we used ZDF rats as a T2DM animal model to identify candidate molecules contributing to T2DM pathogenesis.

T2DM is a complicated disease with interactions among several tissues, such as the liver, muscle, adipose tissue, and pancreas. Thus, diverse anti-diabetic therapeutic strategies have been developed based on the target tissues driving T2DM. Differentially expressed genes (DEGs) involved in the onset of T2DM in insulin-sensitive and insulin-producing tissues from anti-diabetic drug-treated animals have been reported to understand the molecular mechanisms involved in T2DM pathogenesis [4], [6], [14], [16]. However, a comparison of global gene expression changes among animals treated with several anti-diabetic drugs has not been reported. Therefore, we evaluated complicated gene expression profile changes in insulin-sensitive tissues from anti-diabetic drug-treated diabetic rats. We were able to predict functional pathway alterations and gene interactions associated with drug-mediated recovery of T2DM.

## Materials and Methods

### Laboratory animals and drug treatment

Twelve week-old male ZDF (fa/fa) rats were used (Genetic Models Inc., Indianapolis, IN). Age and sex-matched ZLC rats (fa/+) were used as non-diabetic controls. The animals were maintained at 24±2°C with 12 hours of light per day and fed a powdered standard chow diet (Lab Diets, Indianapolis, IN) *ad libitum* with tap water. These procedures were reviewed according to the “Guide for Animal Experiments” (edited by the Korean Academy of Medical Sciences) by the Institutional Animal Care and Use Committee at Seoul National University. The animal protocol was approved by the committee on the Ethics of Animal Experiments of the Seoul National University (Permit Number: SNU-200909-40). All of the experiments were conducted to minimize the number of animals used. Metformin (Glucophage, 300 mg/kg/day) and glimepiride (Amaryl, 200 mg/kg/day) were administered by oral gavage to 12 weeks old diabetic ZDF rats for 2 weeks in each group and rosiglitazone (Avandia, BRL 49653, 15 mg/kg/day) was administered to ZDF rats for 1 week according to the manufacturer's protocol. Five rats were used in each group. On the last day of the experiment, the rats were sacrificed under ether anesthesia and the white adipose tissue from epididymal fat, liver, gastrocnemius muscle, and pancreas were removed and stored at −70°C for further investigation.

### Oral glucose tolerance test (OGTT)

Rats were fasted overnight prior to the OGTT. Glucose (1.0 g/kg body weight) was orally administered to rats with a feeding syringe. Blood samples were collected from the tail vein, and plasma glucose levels were measured immediately before and 15, 30, 45, 60, and 120 min after glucose loading. Blood glucose levels were determined using the SureStep commercial blood glucose meter (Lifescan Co, Milpitas, CA).

### Total RNA isolation

Total RNA was extracted from the three different tissues after drug treatment using Trizol reagent (Invitrogen, Carlsbad, CA) according to the manufacturer's instructions. Isolated RNA was considered pure if the ratio of absorbance readings at 260 and 280 nm was 1.7–2.1. Three rats per groups were used for the microarray experiments.

### Oligonucleotide microarray analysis

The oligonucleotide microarray analysis was performed on the Rat Genome OpArray 27 K (Operon, Ebersberg, Germany). The cDNA of ZDF rats was labeled with Cy3, and the cDNAs of ZLC and anti-diabetic drug-treated ZDF rats were labeled with Cy5. Twelve microarray slides (three rats in each group) were used to monitor gene expression levels. We used Superscript TMll (Invitrogen, Carlsbad, CA) RNase H Reverse Transcriptase to synthesize cDNA. The reverse transcription mixture included 400 U Superscript RNase H-reverse transcriptase, 15 mM dATP, dTTP, and dGTP; 0.6 mM dCTP; and 3 mM Cy3 or Cy5 labeled dCTP (NEN Life Science Product Inc., Boston, MA). After reverse transcription, the sample RNA was degraded by adding 5 µl stop solution (0.5 M NaOH/50 mM EDTA) followed by a 10 min incubation at 65°C. The labeled cDNA mixture was then concentrated using the ethanol precipitation method. The microarrays were washed anhybridized according to the manufacturer's instructions. In brief, the two labeled cDNA probe solutions containing hybridization buffer (Genocheck, Seoul, Korea) were mixed in equal amounts, the HybriWell FL Sealing System (Grace Bio-labs, Bend, OR) was applied to the microarrays, and the microarrays were placed in a humidified chamber at 62°C for 14 h. Then, the microarrays were sequentially washed for 5 min each, once at 65°C in 2× SSC and 0.1% SDS, once in 1× SSC at room temperature, and once in 0.2× SSC at room temperature. Then, the slides were centrifuged for 2 min at 800 rpm to dry the slides.

### Microarray data analysis and DEG network construction

All microarray data was analyzed with R software (http://www.r-project.org/). The expression data were normalized with the print-tip loess method. DEGs between ZDF and the treated samples were identified at an estimated p-value using the Limma package [19]. A dendrogram was constructed by Pearson's correlation and the complete linkage method. The gene interaction network was visualized with Cytoscape Version 2.8.1: An Open Source Platform for Complex Network Analysis and Visualization [20]. The nodes in the network were the DEGs between the ZDF and treated samples. We determined the interaction (edges) between DEGs using Human Net. Because of the poorly annotated rat pathway, we used human gene interaction information with ID conversion through the official symbol. Human Net [21] is a probabilistic functional gene network of validated protein-encoding genes for *Homo sapiens* constructed by modified Bayesian integration of 21 types of omics data. The sub-network was obtained using Cytomcl, which is a plug-in built for Cytoscape [20]. We performed a KEGG [22] pathway enrichment analysis using the Database for Annotation, Visualization and Integrated Discovery 6.7 functional annotation tool (DAVID) to obtain the biological meaning of the sub-networks [23]. The data were deposited in the NCBI Gene Expression Omnibus (GEO) and are accessible through GEO series accession number GSE36714.

### Quantitative real-time polymerase chain reaction (PCR) analysis

Total RNA was isolated using the Total RNA Purification System (Invitrogen, Carlsbad, CA) following the manufacturer's protocol. Quantitative real time PCR was performed with SYBR Green dye using the StepOnePlus™ Real-Time PCR System (Applied Biosystems, Cheshire, U.K.). We used the comparative Ct method (ΔΔCt) to quantify relative gene expression. The results were normalized to the rat β-actin signal. See Table S3 for the complete primer sequences used in the real-time PCR.

### Statistical analysis

Results were expressed as the means ± SEM. Student's t-tests was used to analyze gene expression differences between ZDF and ZLC or rosiglitazone-treated ZDF group in RT-qPCR. Differences were considered significant at *p*<0.05.

## Results

### Effects of anti-diabetic drugs on glucose tolerance in ZDF rats

**Figure 1:**
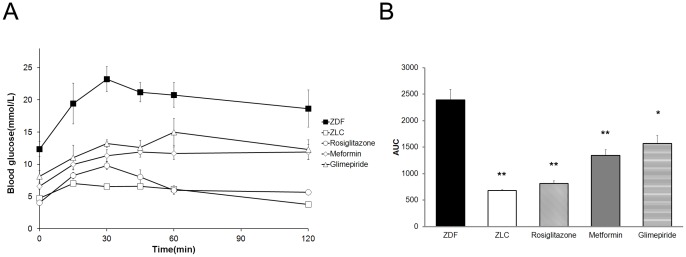
Effects of anti-diabetic drugs on the oral glucose tolerance test (OGTT) in ZDF rats. (A) OGTT in diabetic ZDF rat (▪, black squares), normal ZLC rats (□, white squares), rosiglitazone (○, white circles), metformin (◊, white diamonds) and glimepiride (Δ, white triangles)-treated diabetic ZDF rats. (B) The glucose area under the curve (AUC) during the course of the experiments was calculated. * *p*<0.05, ** *p*<0.01.

Metformin (300 mg/kg/day) and glimepiride (200 mg/kg/day) were administered to 12 week-old ZDF rats for 2 weeks and rosiglitazone (15 mg/kg/day) was administered for 1 week to evaluate the effect of anti-diabetic drugs on impaired glucose tolerance in ZDF rats [24]–[26]. The oral glucose tolerance test (OGTT) was performed on the last day of experiments. Blood glucose levels were significantly higher in the ZDF groups at all time-points during the OGTT, and the area under the curve (AUC) also increased significantly compared to that in normal lean ZLC rats**.** Treating ZDF rats with anti-diabetic drugs significantly reduced blood glucose levels during the OGTT. In addition, the blood glucose AUC was smaller in treated diabetic animals compared with that in control ZDF rats (Fig. 1A, B).

### Gene expression profiles in anti-diabetic drug treated ZDF rats

**Figure 2:**
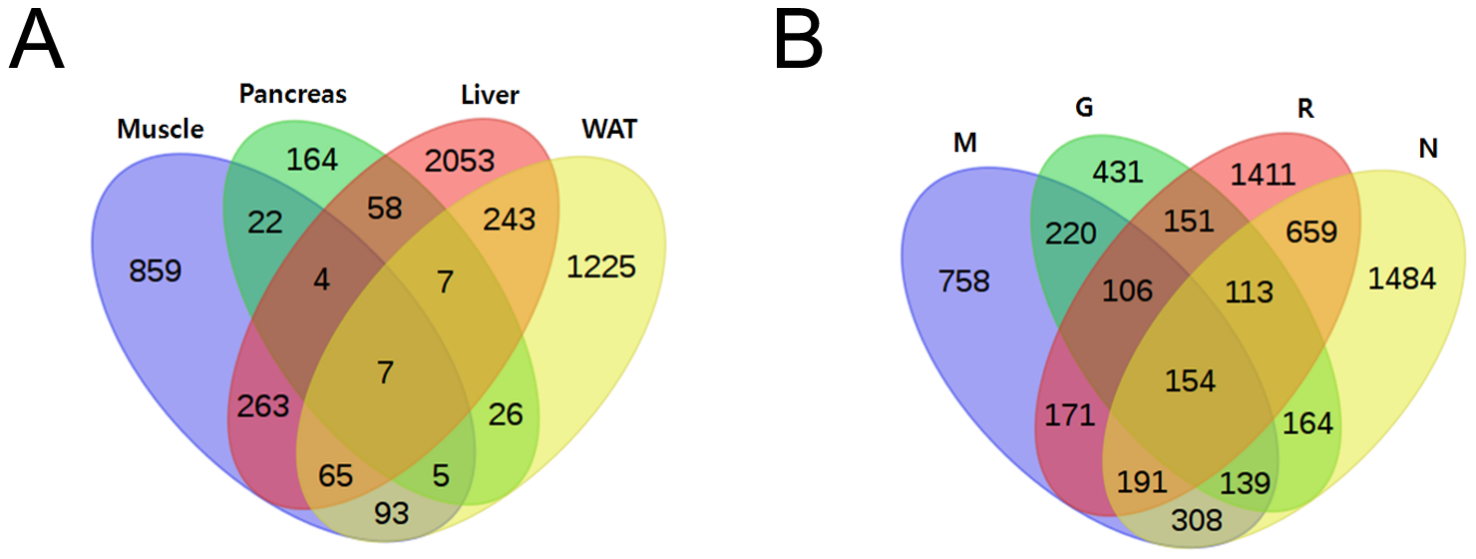
A Venn- diagram showing overlap of differentially expressed gene by multiple group comparison. (A) Comparison between drug conditions with each tissue condition (B) comparison between tissue conditions with each drug condition. Significant differentially expressed genes (*p* value <0.01) are given for each normal ZLC group and the rosiglitazone, glimepiride, and metformin- treated groups.

We investigated multiple anti-diabetic drug therapies for ZDF rats to identify global gene expression changes induced by the diabetic drugs. Twelve- week old ZLC male rats and 12- week old ZDF rats serving as controls were used to study diabetic changes, whereas 12- week old ZDF male rats and age-matched anti-diabetic drug-treated ZDF rats were used to evaluate the diabetic changes induced by the anti-diabetic drugs using oligonucleotide chips containing 27 k rat genes. Three ZLC and anti-diabetic drug treated ZDF rats in each group were used. We performed microarray experiments three times in each experimental group to obtain more accurate and confident results. We determined the DEGs (limma, *p*<0.05) in all experimental groups using the multi class method. The Venn- diagrams presented in Fig. 2 display how many DEGs overlapped under each condition.

**Figure 3:**
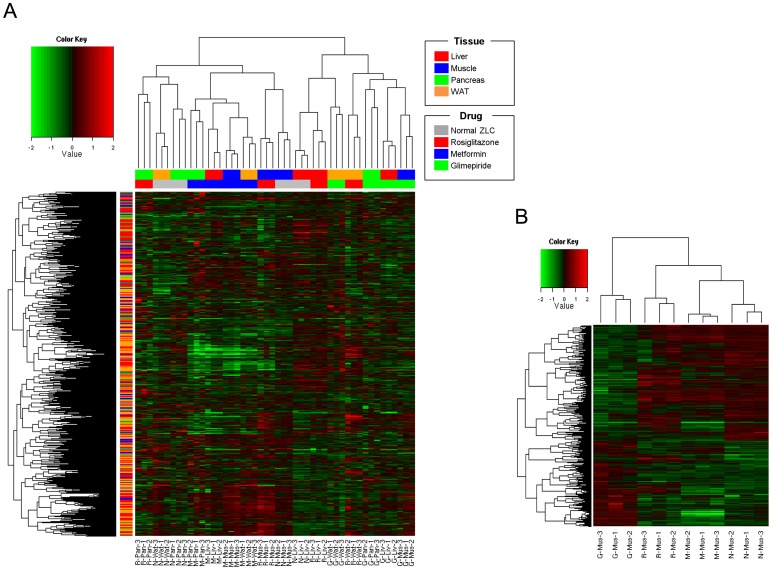
Hierarchical clustering analysis. (A) Expression heat-map with 5,650 differentially expressed genes (DEGs) (excluding the common response DEGs) in all 48 samples. The first row of the color legend under the hierarchical clustering by column indicates sample's tissue type, and the second is the drug type. Another color legend on the side identifies the drug target information. (B) Expression heat-map with 857 genes in 12 muscle samples. (*p* value <0.05 and remove common DEGs and drug-response DEGs) G, Glimepiride; M, Metformin; R, Rosiglitazone; N, normal ZLC.

Hierarchical clustering was constructed for the DEGs to show the multi-drug effect in four tissues. Hierarchical clustering showed that groups under the same experimental conditions closely aligned. Each anti-diabetic drug treated sample was similarly clustered with normal samples in the target tissue (Fig. 3A). Rosiglitazone improves glucose metabolism mainly by suppressing hepatic glucose production and accelerating glucose utilization in white adipose tissue [8]. Rosiglitazone-treated samples clustered together with normal samples in liver, suggesting that rosiglitazone treatment normalized hepatic gene expression changes associated with T2DM **(** **Fig. 3A** **)**. Similarly, the metformin treated samples were the nearest neighbor of normal muscle samples which is a known target tissue.

### Integrated DEG network

**Figure 4:**
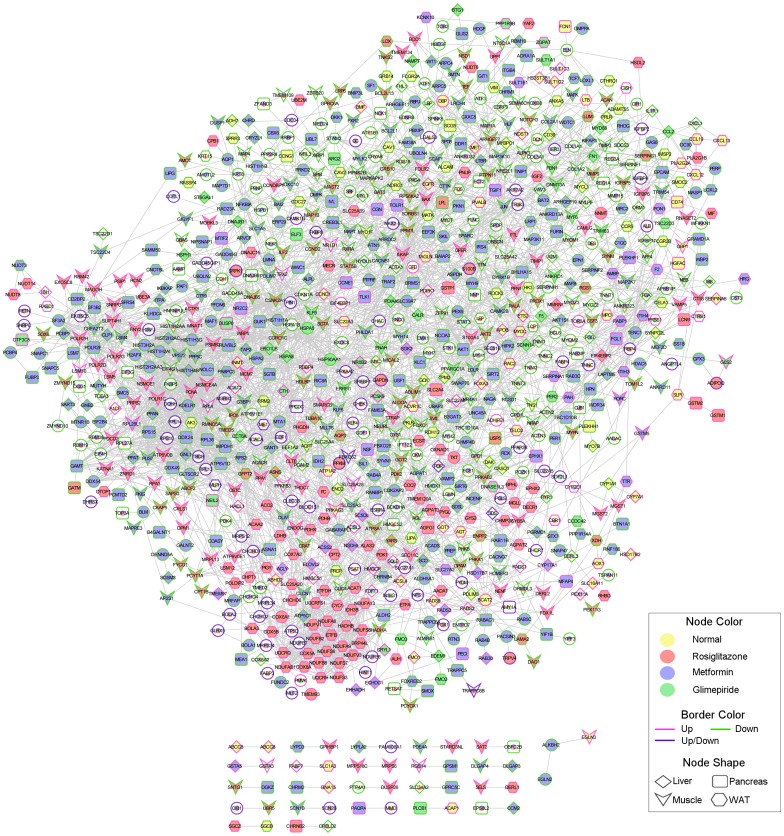
Gene-gene interaction network among the experimental groups. An integrated network was generated by the Human Net database. Edges represent interactions between significantly expressed genes.

**Figure 5:**
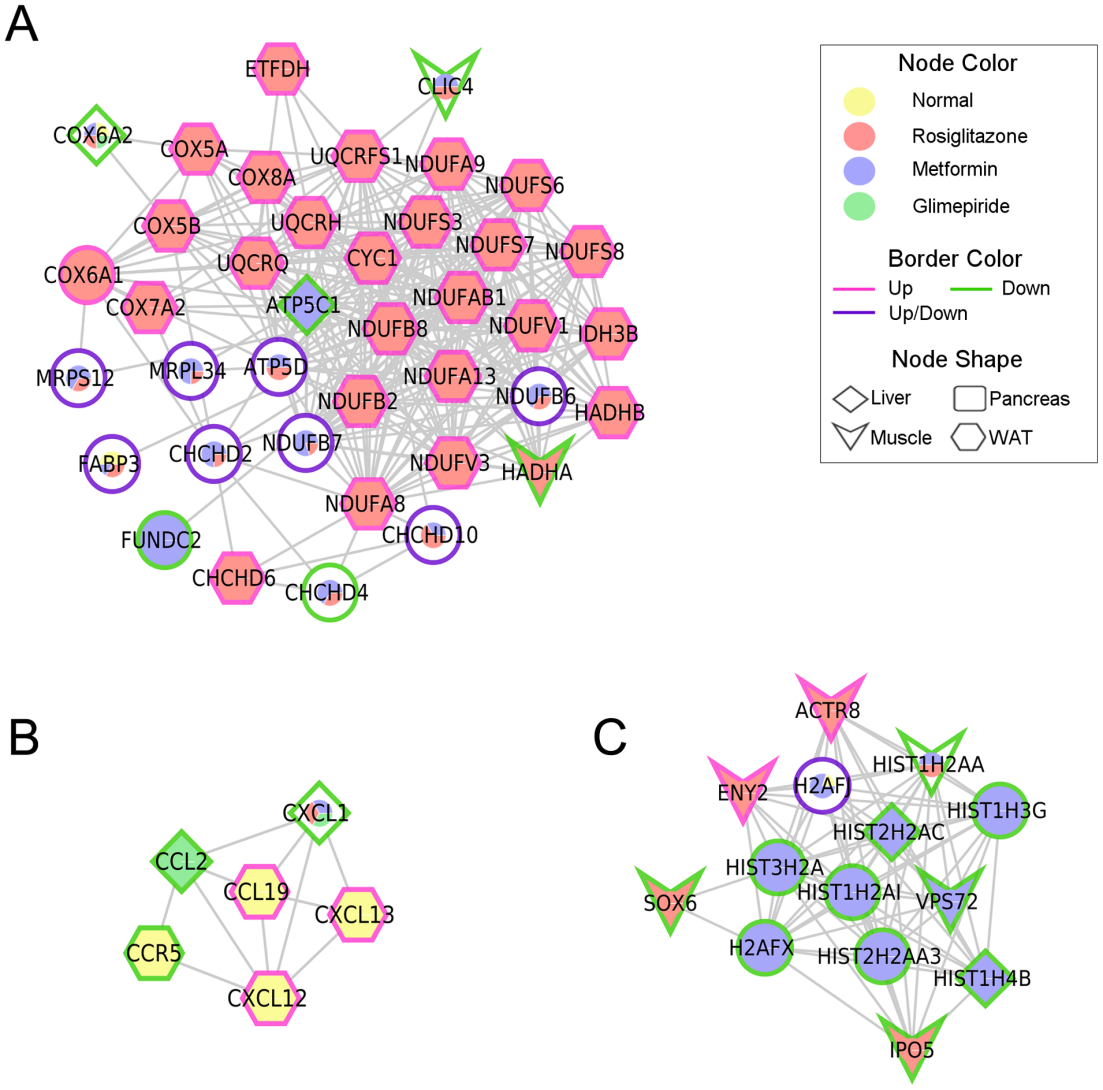
Selected enriched sub-networks. The color of the pie chart in the node illustrates the proportion of treatment condition and the angle size is the observed frequency of differentially expressed genes (DEGs) for specific drugs in the four tissues. Border color indicates expression patterns. Node shape is tissue type. Enriched pathway: (A) oxidative phosphorylation, (B) chemokine signaling pathway, (C) systemic lupus erythematosus.

To predict the drug effect in a specific tissue, we conducted a functional annotation network analysis with the DEGs from the anti-diabetic drug treatments. DEGs between ZDF and the treated samples were identified by a p- value ≤0.01 and |log2 FC| >2. A total of 680, 913, 749, and 554 genes changed in liver, muscle, white adipose tissue, and pancreas, respectively. We focused on the common DEGs between normal ZLC rats and the three anti-diabetic drug groups, as these genes might be regulated by anti-diabetic drugs and involved in diabetes resistance. A total of 81 subnetworks were obtained through this analysis (Fig. 4, Table S1). Among them, several biological pathways were significantly enriched by the anti-diabetic drugs, including OXPHOS, systemic lupus erythematous, and the chemokine signaling pathway (Fig. 5, Table S2). Twenty-three genes involved in OXPHOS were up-regulated in white adipose tissue of rosiglitazone-treated ZDF rats. Furthermore, genes related to the systemic lupus erythematous pathway were significantly down-regulated in muscle and liver of metformin-treated ZDF rats. Three genes involved in the chemokine signaling pathway were up-regulated in white adipose tissue of ZLC rats, whereas the other three genes were down-regulated by the anti-diabetic drugs.

### Validation by real-time polymerase chain reaction (PCR)

**Figure 6:**
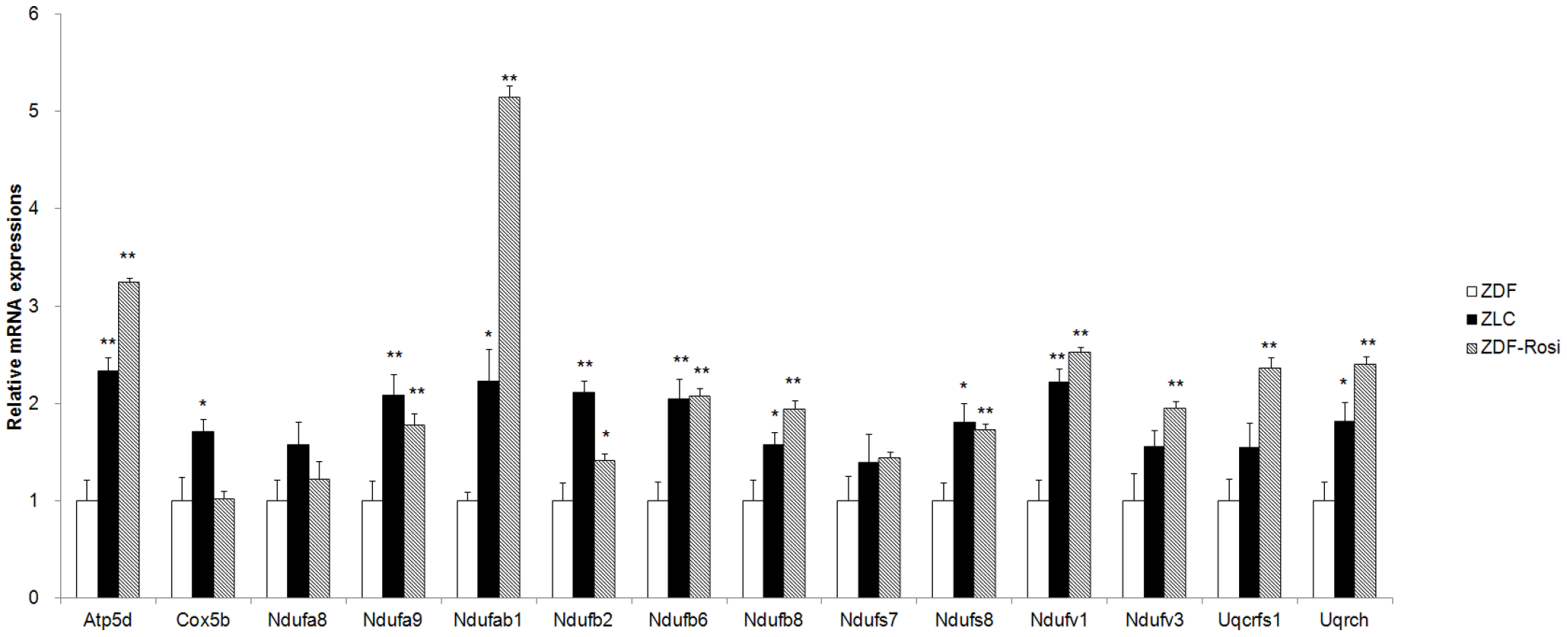
Evaluation of oxidative phosphorylation-related genes transcription. Quantitative polymerase chain reaction (PCR) mRNA expression for genes involved in oxidative phosphorylation in white adipose tissue of ZLC or ZDF rats treated with rosiglitazone. * *p*<0.05, ** *p*<0.01.

Real-time PCR was performed on a subset of 14 genes involved in OXPHOS functioning in the white adipose tissues of rosiglitazone treated ZDF, normal ZLC, and control ZDF rats to confirm changes in the microarray data (Fig. 6). As a result, the direction of change obtained by real-time PCR was similar to that observed in the microarray results (ATP5d, NDUFA9, NDUFAB1, NDUFAB2, NDUFAB 6, NDUFAB 8, NDUFS8, NDUFV1, NDUFV3, UQCRFS1, and UQCRH). However, three genes (COX5B, NDUFS7, and NDUFA8) did not change significantly.

## Discussion

We investigated the divergent effect of anti-diabetic drugs on gene expression in 12 week old diabetic ZDF rats using oligonucleotide microarray technology. We used three different kinds of anti-diabetic drugs that have different mechanisms to manage T2DM. Treating the ZDF rats with the anti-diabetic drugs resulted in improved glucose tolerance compared with that in the untreated control ZDF rats. To analyze the changes at the transcriptome level caused by the anti-diabetic drugs, we used multiple group comparison (Multi-Class: The data exists as multiple groups, and differential expression is found between groups.). Although each anti-diabetic drug altered the expression of many genes, only a small number of genes were commonly regulated by all anti-diabetic drugs, which may have been caused by different mechanisms of action (Fig. 2). We hypothesized from the Venn diagram results that each drug may act differently on each tissue with a different function. Thus, we showed the relationship between the drug and target tissue and predicted the target pathway using the DEG expression pattern.

Rosiglitazone is an anti-diabetic drug in the thiazolidinedione (TZD) class. TZDs induce lipid repartitioning by increasing adipose triglyceride (TG) content, thereby lowering circulating free fatty acids, glycerol, TG, and glucose, which are associated with increased insulin sensitivity of the liver, muscle, and other organs [27]–[29]. The hierarchical cluster analysis revealed that the gene expression pattern in the liver of rosiglitazone-treated ZDF rats showed a similar pattern to that of normal liver (Fig. 3A). We conducted a Gene Set Analysis (GSA) with the DEGs from the liver of rats in each of the drug conditions to better understand the varying effects of rosiglitazone on liver. From the results, we found that the peroxisome proliferator-activated receptor (PPAR) signaling and fatty acid metabolism pathways were significantly enriched in the liver of rosiglitazone treated rats (p<0.05, Table S4). These observations suggest that rosiglitazone may regulate PPAR signaling gene expression, which affects lipid metabolism in the liver. These transcriptional changes may also contribute to altered glucose and fatty acid metabolism characteristic of the development of T2DM.

We also found a similar pattern of muscle gene expression in metformin-treated ZDF rats and normal ZLC rats (Fig. 3A). Interestingly, gene expression of each tissue from metformin-treated ZDF rats clustered together. We conducted GSA with the genes commonly regulated by metformin treatment to better understand this result. PPAR signaling-related genes were enriched in up-regulated DEGs and down-regulated DEGs were related to the MAPK signaling pathway and vascular smooth muscle contraction-related genes. Metformin treatment also commonly regulated gene expression related to lipid metabolism including ACOX1 and CPT1a in all tissues (Table S5). AMPK signaling is a known target of metformin action [30], [31]. AMPK enhances fatty acid oxidation through changes in expression of metabolic enzymes including Acox1 and Cpt1a. This result suggests that metformin may affect AMPK gene expression in target tissues such as ACOX1 and CPT1a.

We found that rosiglitazone and metformin treatment clustered with normal ZLC muscle when we used hierarchical clustering of muscle tissues only (Fig. 3B). Several studies have established that metformin and rosiglitazone activate AMPK in both hepatocytes and skeletal muscle [32], [33]. We were interested in finding the different target pathways between metformin and rosiglitazone in muscle. Using the DEGs between rosiglitazone and metformin-treated muscle samples, we found different drug effects in muscle tissue. rno05410: the hypertrophic cardiomyopathy (HCM) pathway was enriched in up-regulated DEGs and rno04260: the cardiac muscle contraction pathway was enriched in down-regulated DEGs (p<0.05) (Fig. S1). Metformin treatment reduces the risk of myocardial infarction by approximately 39% [34], [35]. In contrast, use of rosiglitazone is limited because of a potential increased risk for myocardial infarction [36], [37]. Consistent with these findings, our results should be interpreted by considering the side effect of rosiglitazone related to myocardial infarction. Down-regulated TNNC1 [38] and up-regulated titin [38] in rosiglitazone-treated ZDF rats is correlated with increased side effects leading to cardiac problems by rosiglitazone. Our results provide different modes of action between rosiglitazone and metformin and show indirect evidence for increasing the risk of cardiac problem using omics data, but not biological validation.

Glimepiride is a medium- to long-acting sulfonylurea anti-diabetic drug that induces insulin Secretagogues act by binding to the SUR subunit of the ATP-sensitive potassium (KATP) channel [39], [40]. No significant pattern of gene expression in other tissues except the pancreas was observed following glimepiride treatment. The pancreas is the only organ that secretes insulin unlike other insulin-sensitive tissues. Thus, we thought that glimepiride, which improves insulin secretion from the pancreas, might only affect the pancreas.

To predict the drug effect in a specific tissue, we constructed a gene interaction network using genes that were differentially expressed between normal and drug treated tissue. Among 81 subnetworks, we focused on three subnetworks. Most nodes of the first subnetwork, which was significantly enriched with OXPHOS, were DEGs in white adipose tissue of rosiglitazone-treated ZDF rats (Fig. 5A). OXPHOS is the key mechanism for glucose and fatty acid metabolism. Down-regulation of genes involved in OXPHOS has been described in muscle and adipose tissues of patients with T2DM [15], [41]. The OXPHOS pathway protein complex consists of five different subunits, including nicotinamide adenine dinucleotide–ubiquinone oxidoreductase, succinate–ubiquinone oxidoreductase, ubiquinol–cytochrome *c* reductase, cytochrome *c* oxidase, and ATP synthase. We identified 25 genes involved in complexes I–V of the electron transport chain that were up-regulated by rosiglitazone treatment (Fig. 5A, 6). Our result demonstrated that OXPHOS related gene expression is down-regulated and might impair oxidative metabolism in white adipose tissues from diabetic ZDF rats but was restored by rosiglitazone treatment.

Chronic inflammation is associated with insulin resistance. Chemokines and proinflammatory stimuli activate the nuclear factor-κB and JNK pathways in insulin sensitive tissues and may cause insulin resistance [42]. CCL2, also called chemokine monocyte chemotactic protein-1, impairs adipocyte insulin sensitivity [43]. Anti-diabetic drugs treatment induced down-regulation of CCL2 and CXCL1 gene expression in liver (Fig. 5B). Our result shows that treatment of anti-diabetic drugs reduced gene expression involved in chronic inflammatory genes and may improve the chronic inflammatory status in liver of diabetic ZDF rats.

Systemic lupus erythematosus (SLE) is a systemic autoimmune disease that can influence any part of body and often coexists with other diseases. The association between inflammatory diseases such as SLE or rheumatoid arthritis and metabolic syndrome has been reported [44]. We found that metformin down-regulated gene expression in the liver and muscle related to SLE (Fig. 5C). This result indicates metformin reduce gene expression related to SLE and might prevent diabetes related disease status.

Taken together, we found that dramatic gene expression changes were induced by each anti-diabetic drug treatment in insulin sensitive tissues of ZDF rats using the microarray technique. Changes in gene expression profiles associated with OXPHOS, SLE, and chemokine signaling related genes were found in insulin sensitive tissue from anti-diabetic drug- treated ZDF rats. In particular, we found decreased expression of many OXPHOS genes in white adipose tissue of diabetic ZDF rats which was normalized with decreasing blood glucose level by rosiglitazone treatment. Through the comprehensive gene expression network study from a multi-drug treatment on ZDF rats, we suggest that alterations in OXPHOS genes expression in white adipose tissue may play a role in the pathogenesis and anti-diabetic drug-mediated recovery of T2DM.

## Supporting Information

Figure S1 **Adverse effect of rosiglitazone treatment.** GSA performed with 2,769 differentially expressed genes (DEGs) from muscle of rosiglitazone and metformin-treated ZDF rats. Data were analyzed using the KEGG pathway feature in DAVID software. Red stars designate DEG of the pathway present. (A) Up-regulated genes significantly related to the hypertrophic cardiomyopathy (HCM) pathway (*p*<0.05). (B) Down-regulated genes significantly related to cardiac muscle contraction pathway.
(TIF)

Table S1 **List of subnetworks.**
(DOCX)

Table S2 **List of genes from selected subnetworks.**
(DOCX)

Table S3 **Sequences of the rat real-time primer pairs used.**
(DOCX)

Table S4 **GSA results from total intersect.**
(ZIP)

Table S5 **GSA with the DEGs commonly regulated by metformin treatment.**
(DOCX)
