# Supplementary figures and images for: Transcriptomic Analysis of Insulin-Sensitive Tissues from Anti-Diabetic Drug Treated ZDF Rats, a T2DM Animal Model
Source: PLoS One. 2013 Jul 26;8(7):e69624. doi: 10.1371/journal.pone.0069624 (PMC3724940; doi:10.1371/journal.pone.0069624)

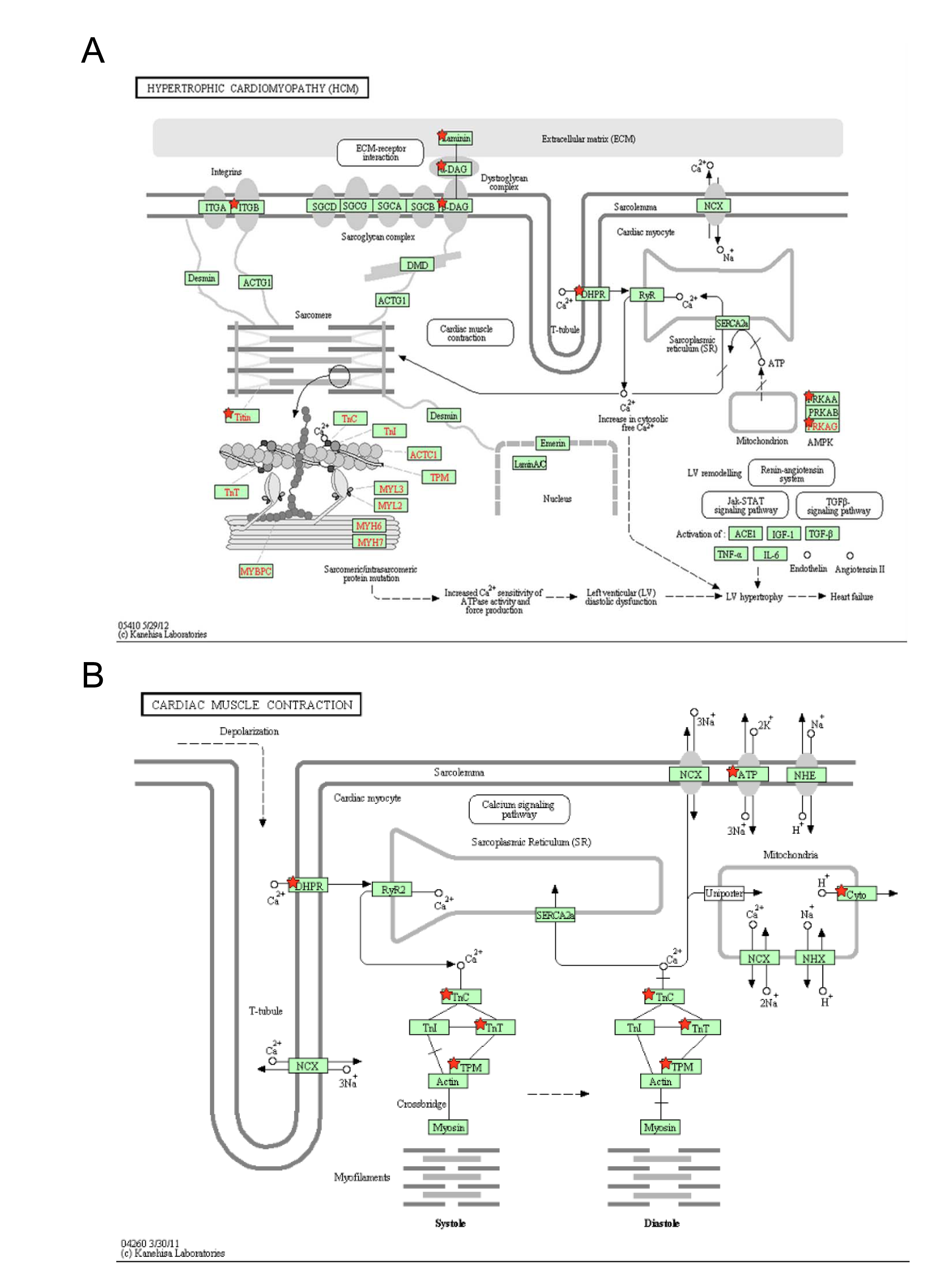

Supplement: Figure S1 — Adverse effect of rosiglitazone treatment. GSA performed with 2,769 differentially expressed genes (DEGs) from muscle of rosiglitazone and metformin-treated ZDF rats. Data were analyzed using the KEGG pathway feature in DAVID software. Red stars designate DEG of the pathway present. (A) Up-regulated genes significantly related to the hypertrophic cardiomyopathy (HCM) pathway (p<0.05). (B) Down-regulated genes significantly related to cardiac muscle contraction pathway. (TIF) [file pone.0069624.s001.tif]
