# Supplementary material for: Transcriptomic Analysis of Insulin-Sensitive Tissues from Anti-Diabetic Drug Treated ZDF Rats, a T2DM Animal Model
Source: PLoS One. 2013 Jul 26;8(7):e69624. doi: 10.1371/journal.pone.0069624 (PMC3724940; doi:10.1371/journal.pone.0069624)
Supplement: Table S1 — List of subnetworks. (DOCX) [file pone.0069624.s002.docx]

**Table S1. List of subnetworks**

| Motif | Total | Hit | Path | *p*-value | Gene |
| --- | --- | --- | --- | --- | --- |
| 1 | 27 | 25 | hsa05012:Parkinson's disease | <10^-3^ | ATP5D, NDUFB6, NDUFB7, NDUFB8, CYC1, NDUFAB1, COX5A, UQCRFS1, COX5B, UQCRQ, NDUFB2, NDUFS7, NDUFS6, NDUFS8, NDUFS3, COX7A2, NDUFA8, NDUFA9, COX8A, NDUFV3, UQCRH, NDUFV1, COX6A2, ATP5C1, COX6A1 |
| 1 | 27 | 25 | hsa00190:Oxidative phosphorylation | <10^-3^ | ATP5D, NDUFB6, NDUFB7, NDUFB8, CYC1, NDUFAB1, COX5A, UQCRFS1, COX5B, UQCRQ, NDUFB2, NDUFS7, NDUFS6, NDUFS8, NDUFS3, COX7A2, NDUFA8, NDUFA9, COX8A, NDUFV3, UQCRH, NDUFV1, COX6A2, ATP5C1, COX6A1 |
| 1 | 27 | 25 | hsa05010:Alzheimer's disease | <10^-3^ | ATP5D, NDUFB6, NDUFB7, NDUFB8, CYC1, NDUFAB1, COX5A, UQCRFS1, COX5B, UQCRQ, NDUFB2, NDUFS7, NDUFS6, NDUFS8, NDUFS3, COX7A2, NDUFA8, NDUFA9, COX8A, NDUFV3, UQCRH, NDUFV1, COX6A2, ATP5C1, COX6A1 |
| 1 | 27 | 25 | hsa05016:Huntington's disease | <10^-3^ | ATP5D, NDUFB6, NDUFB7, NDUFB8, CYC1, NDUFAB1, COX5A, UQCRFS1, COX5B, UQCRQ, NDUFB2, NDUFS7, NDUFS6, NDUFS8, NDUFS3, COX7A2, NDUFA8, NDUFA9, COX8A, NDUFV3, UQCRH, NDUFV1, COX6A2, ATP5C1, COX6A1 |
| 1 | 27 | 10 | hsa04260:Cardiac muscle contraction | <10^-3^ | COX7A2, UQCRH, CYC1, COX8A, COX6A2, COX6A1, UQCRFS1, COX5A, UQCRQ, COX5B |
| 1 | 27 | 2 | hsa00062:Fatty acid elongation in mitochondria | 0.043 | HADHA, HADHB |
| 2 | 10 | 10 | hsa03010:Ribosome | <10^-3^ | MRPL13, RPL14, RPL13, RPS15, RPL27A, RPL26L1, RPL36, RPS10, RPL4, RPS2 |
| 3 | 13 | 9 | hsa03040:Spliceosome | <10^-3^ | SFRS4, MAGOH, LSM7, LSM5, LSM4, SNRPC, SF3A2, SNRPG, SF3B2 |
| 3 | 13 | 4 | hsa03020:RNA polymerase | <10^-3^ | POLR2H, POLR2G, POLR2E, POLR2D |
| 3 | 13 | 4 | hsa00240:Pyrimidine metabolism | 0.001 | POLR2H, POLR2G, POLR2E, POLR2D |
| 3 | 13 | 4 | hsa00230:Purine metabolism | 0.005 | POLR2H, POLR2G, POLR2E, POLR2D |
| 3 | 13 | 4 | hsa05016:Huntington's disease | 0.008 | POLR2H, POLR2G, POLR2E, POLR2D |
| 3 | 13 | 3 | hsa03018:RNA degradation | 0.008 | LSM7, LSM5, LSM4 |
| 4 | 3 | 3 | hsa04610:Complement and coagulation cascades | 0.003 | FGG, FGA, F5 |
| 5 | 6 | 6 | hsa04512:ECM-receptor interaction | <10^-3^ | CD36, COL3A1, COL1A2, COL2A1, COL1A1, FN1 |
| 5 | 6 | 5 | hsa04510:Focal adhesion | <10^-3^ | COL3A1, COL1A2, COL2A1, COL1A1, FN1 |
| 6 | 9 | 9 | hsa05322:Systemic lupus erythematosus | <10^-3^ | HIST2H2AA3, HIST1H2AA, HIST1H4B, HIST2H2AC, HIST1H2AI, HIST3H2A, H2AFX, H2AFJ, HIST1H3G |
| 7 | 3 | 3 | hsa04612:Antigen processing and presentation | 0.001 | HSPA2, HSPA1B, HSPA8 |
| 7 | 3 | 3 | hsa03040:Spliceosome | 0.002 | HSPA2, HSPA1B, HSPA8 |
| 7 | 3 | 3 | hsa04144:Endocytosis | 0.004 | HSPA2, HSPA1B, HSPA8 |
| 7 | 3 | 3 | hsa04010:MAPK signaling pathway | 0.008 | HSPA2, HSPA1B, HSPA8 |
| 9 | 8 | 6 | hsa00500:Starch and sucrose metabolism | <10^-3^ | PYGM, PYGL, GYS1, GYS2, AGL, AMY1A |
| 9 | 8 | 6 | hsa04910:Insulin signaling pathway | <10^-3^ | PYGM, PHKB, PYGL, PHKA1, GYS1, GYS2 |
| 12 | 2 | 2 | hsa04144:Endocytosis | 0.036 | RAB5C, RAB11B |
| 16 | 2 | 2 | hsa05014:Amyotrophic lateral sclerosis (ALS) | 0.010 | BAX, BCL2L1 |
| 16 | 2 | 2 | hsa04210:Apoptosis | 0.017 | BAX, BCL2L1 |
| 18 | 5 | 3 | hsa00620:Pyruvate metabolism | 0.001 | PKLR, ACACA, PC |
| 18 | 5 | 3 | hsa00010:Glycolysis / Gluconeogenesis | 0.002 | PKLR, ENO2, PFKM |
| 19 | 2 | 2 | hsa05412:Arrhythmogenic right ventricular cardiomyopathy (ARVC) | 0.030 | ACTN2, ACTN3 |
| 19 | 2 | 2 | hsa04520:Adherens junction | 0.030 | ACTN2, ACTN3 |
| 19 | 2 | 2 | hsa05322:Systemic lupus erythematosus | 0.039 | ACTN2, ACTN3 |
| 19 | 2 | 2 | hsa04670:Leukocyte transendothelial migration | 0.046 | ACTN2, ACTN3 |
| 21 | 4 | 3 | hsa00020:Citrate cycle (TCA cycle) | <10^-3^ | ACLY, DLAT, PDHB |
| 21 | 4 | 3 | hsa00620:Pyruvate metabolism | 0.001 | DLAT, ACACB, PDHB |
| 23 | 4 | 4 | hsa03320:PPAR signaling pathway | <10^-3^ | SLC27A1, ACSL4, ACSL3, SLC27A2 |
| 23 | 4 | 2 | hsa00071:Fatty acid metabolism | 0.046 | ACSL4, ACSL3 |
| 30 | 7 | 3 | hsa00190:Oxidative phosphorylation | 0.009 | ATP6V1D, ATP6V0B, PPA1 |
| 30 | 7 | 2 | hsa00020:Citrate cycle (TCA cycle) | 0.036 | ACO2, IDH2 |
| 30 | 7 | 2 | hsa00071:Fatty acid metabolism | 0.046 | ACAA2, ACAT2 |
| 31 | 2 | 2 | hsa04020:Calcium signaling pathway | 0.035 | TNNC2, TNNC1 |
| 32 | 2 | 2 | hsa00900:Terpenoid backbone biosynthesis | 0.015 | HMGCR, HMGCS1 |
| 34 | 3 | 3 | hsa01040:Biosynthesis of unsaturated fatty acids | <10^-3^ | FADS1, SCD, FADS2 |
| 34 | 3 | 2 | hsa03320:PPAR signaling pathway | 0.027 | SCD, FADS2 |
| 38 | 6 | 5 | hsa00980:Metabolism of xenobiotics by cytochrome P450 | <10^-3^ | CYP1A1, CYP2E1, GSTM5, MGST1, MGST2 |
| 38 | 6 | 4 | hsa00982:Drug metabolism | <10^-3^ | CYP2E1, GSTM5, MGST1, MGST2 |
| 38 | 6 | 3 | hsa00480:Glutathione metabolism | 0.001 | GSTM5, MGST1, MGST2 |
| 38 | 6 | 2 | hsa00140:Steroid hormone biosynthesis | 0.044 | CYP1A1, CYP7A1 |
| 39 | 6 | 6 | hsa04062:Chemokine signaling pathway | <10^-3^ | CXCL1, CCL2, CCR5, CXCL13, CCL19, CXCL12 |
| 39 | 6 | 6 | hsa04060:Cytokine-cytokine receptor interaction | <10^-3^ | CXCL1, CCL2, CCR5, CXCL13, CCL19, CXCL12 |
| 40 | 2 | 2 | hsa00240:Pyrimidine metabolism | 0.037 | NT5C1A, UPP1 |
| 43 | 3 | 2 | hsa00640:Propanoate metabolism | 0.025 | ALDH2, ACSS2 |
| 43 | 3 | 2 | hsa00650:Butanoate metabolism | 0.026 | HMGCS2, ALDH2 |
| 43 | 3 | 2 | hsa00620:Pyruvate metabolism | 0.031 | ALDH2, ACSS2 |
| 43 | 3 | 2 | hsa00280:Valine, leucine and isoleucine degradation | 0.034 | HMGCS2, ALDH2 |
| 43 | 3 | 2 | hsa00010:Glycolysis / Gluconeogenesis | 0.046 | ALDH2, ACSS2 |
| 45 | 5 | 5 | hsa00561:Glycerolipid metabolism | <10^-3^ | GPAM, AGPAT3, PPAP2B, AGPAT2, AGPAT1 |
| 45 | 5 | 5 | hsa00564:Glycerophospholipid metabolism | <10^-3^ | GPAM, AGPAT3, PPAP2B, AGPAT2, AGPAT1 |
| 45 | 5 | 4 | hsa00565:Ether lipid metabolism | <10^-3^ | AGPAT3, PPAP2B, AGPAT2, AGPAT1 |
| 50 | 2 | 2 | hsa00592:alpha-Linolenic acid metabolism | 0.007 | PLA2G1B, PLA2G2A |
| 50 | 2 | 2 | hsa00591:Linoleic acid metabolism | 0.011 | PLA2G1B, PLA2G2A |
| 50 | 2 | 2 | hsa00565:Ether lipid metabolism | 0.014 | PLA2G1B, PLA2G2A |
| 50 | 2 | 2 | hsa00590:Arachidonic acid metabolism | 0.022 | PLA2G1B, PLA2G2A |
| 50 | 2 | 2 | hsa00564:Glycerophospholipid metabolism | 0.027 | PLA2G1B, PLA2G2A |
| 50 | 2 | 2 | hsa04730:Long-term depression | 0.027 | PLA2G1B, PLA2G2A |
| 50 | 2 | 2 | hsa04370:VEGF signaling pathway | 0.029 | PLA2G1B, PLA2G2A |
| 50 | 2 | 2 | hsa04664:Fc epsilon RI signaling pathway | 0.030 | PLA2G1B, PLA2G2A |
| 50 | 2 | 2 | hsa04912:GnRH signaling pathway | 0.038 | PLA2G1B, PLA2G2A |
| 50 | 2 | 2 | hsa04270:Vascular smooth muscle contraction | 0.044 | PLA2G1B, PLA2G2A |
| 53 | 2 | 2 | hsa00920:Sulfur metabolism | 0.005 | SULT1A1, SULT1E1 |
| 59 | 2 | 2 | hsa04810:Regulation of actin cytoskeleton | 0.042 | ROCK2, RDX |
| 60 | 3 | 3 | hsa05416:Viral myocarditis | 0.001 | MYH1, MYH2, MYH7 |
| 60 | 3 | 3 | hsa04530:Tight junction | 0.002 | MYH1, MYH2, MYH7 |
| 64 | 2 | 2 | hsa00564:Glycerophospholipid metabolism | 0.027 | GPD1L, GPD1 |
| 75 | 2 | 2 | hsa00052:Galactose metabolism | 0.005 | GCK, HK3 |
| 75 | 2 | 2 | hsa00500:Starch and sucrose metabolism | 0.008 | GCK, HK3 |
| 75 | 2 | 2 | hsa00520:Amino sugar and nucleotide sugar metabolism | 0.009 | GCK, HK3 |
| 75 | 2 | 2 | hsa04930:Type II diabetes mellitus | 0.009 | GCK, HK3 |
| 75 | 2 | 2 | hsa00010:Glycolysis / Gluconeogenesis | 0.012 | GCK, HK3 |
| 75 | 2 | 2 | hsa04910:Insulin signaling pathway | 0.027 | GCK, HK3 |
| 81 | 3 | 3 | hsa04630:Jak-STAT signaling pathway | 0.001 | PRLR, SOCS3, CISH |
