# Supplementary material for: Transcriptomic Analysis of Insulin-Sensitive Tissues from Anti-Diabetic Drug Treated ZDF Rats, a T2DM Animal Model
Source: PLoS One. 2013 Jul 26;8(7):e69624. doi: 10.1371/journal.pone.0069624 (PMC3724940; doi:10.1371/journal.pone.0069624)
Supplement: Table S2 — List of genes from selected subnetworks. (DOCX) [file pone.0069624.s003.docx]

**Table S2. List of genes from selected subnetworks**

| ID | N-Liv | | G-Liv | R-Liv | M-Liv | N-Mus | G-Mus | R-Mus | M-Mus | N-Pan | G-Pan | R-Pan | M-Pan | N-Wat | G-Wat | R-Wat | M-Wat |
| --- | --- | --- | --- | --- | --- | --- | --- | --- | --- | --- | --- | --- | --- | --- | --- | --- | --- |
| Oxidative phosphorylation | | | |  |  |  |  |  |  |  |  |  |  |  |  |  |  |
| ATP5C1 | -0.2 | | 0.0 | -0.2 | -1.1 | 0.0 | 0.0 | 0.0 | -0.2 | -0.1 | -0.1 | 0.0 | 0.3 | 0.1 | 0.2 | 0.6 | 0.0 |
| ATP5D | -0.1 | | 0.1 | 0.6 | -1.4 | -0.2 | 0.3 | -1.1 | -1.8 | 0.1 | 0.3 | -1.2 | -1.7 | 0.0 | 0.3 | 1.5 | -0.9 |
| COX5A | -0.2 | | 0.1 | 0.1 | -0.1 | 0.0 | -0.3 | -0.1 | -0.4 | -0.1 | -0.1 | -0.1 | -0.2 | -0.2 | -0.1 | 1.1 | 0.2 |
| COX5B | -0.4 | | 0.1 | 0.0 | -0.6 | 0.0 | -0.2 | -0.2 | -0.6 | 0.2 | 0.0 | 0.0 | -0.7 | -0.2 | -0.2 | 1.2 | 0.1 |
| COX6A1 | -0.2 | | 0.2 | 0.3 | -0.8 | 0.0 | 0.7 | 1.1 | -0.6 | 0.2 | 0.3 | -0.1 | -0.9 | -0.1 | 0.0 | 1.7 | -0.3 |
| COX6A2 | -3.1 | | -2.5 | -2.5 | -2.1 | 0.0 | -0.2 | -0.8 | -0.6 | 0.1 | -0.1 | 1.8 | -0.5 | 1.4 | -0.4 | 0.0 | -0.1 |
| COX7A2 | -0.3 | | 0.1 | -0.3 | 0.2 | 0.0 | 0.5 | 0.8 | 0.2 | 0.1 | -0.2 | 0.6 | 0.5 | 0.0 | -0.1 | 1.1 | 0.6 |
| COX8A | -0.2 | | 0.1 | 0.4 | -0.8 | 0.0 | 0.4 | 0.7 | -0.6 | 0.1 | 0.2 | -0.1 | -0.8 | -0.2 | 0.0 | 1.3 | -0.2 |
| CYC1 | -0.4 | | -0.2 | -0.1 | -0.5 | -0.2 | 0.0 | -0.1 | -0.8 | 0.2 | 0.2 | 0.3 | -0.4 | -0.4 | -0.1 | 1.2 | -0.1 |
| NDUFA8 | -0.3 | | 0.1 | 0.1 | -0.3 | 0.0 | 0.4 | 0.5 | 0.0 | 0.2 | 0.0 | -0.2 | -0.6 | 0.3 | -0.2 | 1.0 | 0.5 |
| NDUFA9 | -0.4 | | 0.0 | -0.2 | 0.1 | 0.0 | -0.1 | 0.0 | -0.5 | 0.1 | 0.0 | 0.4 | 0.4 | 0.3 | 0.0 | 1.0 | 0.7 |
| NDUFAB1 | -0.3 | | 0.1 | -0.1 | -0.2 | -0.1 | 0.4 | 0.3 | 0.2 | -0.2 | -0.1 | 0.2 | 0.0 | 0.0 | -0.1 | 1.3 | 0.5 |
| NDUFB2 | -0.2 | | 0.3 | 0.0 | -0.4 | 0.1 | 0.4 | 0.2 | -0.2 | 0.3 | 0.2 | 0.3 | -0.3 | 0.0 | 0.3 | 1.1 | 0.2 |
| NDUFB6 | -0.2 | | 0.2 | 0.2 | -1.3 | 0.0 | 0.3 | -0.3 | -1.5 | 0.0 | 0.0 | -0.2 | -0.7 | -0.1 | 0.0 | 2.1 | -0.6 |
| NDUFB7 | -0.4 | | 0.0 | 0.3 | -2.2 | -0.1 | 0.4 | -0.3 | -1.7 | 0.3 | 0.1 | -0.7 | -2.2 | 0.3 | 0.1 | 1.9 | -1.2 |
| NDUFB8 | -0.3 | | 0.2 | -0.1 | -0.5 | -0.2 | 0.5 | 0.0 | -0.4 | 0.1 | 0.2 | 0.0 | -0.6 | 0.1 | 0.1 | 1.4 | 0.2 |
| NDUFS3 | -0.5 | | 0.0 | 0.0 | -0.4 | -0.1 | 0.1 | -0.2 | -0.8 | -0.1 | 0.2 | -0.1 | -0.5 | 0.0 | -0.2 | 1.2 | 0.1 |
| NDUFS6 | -0.4 | | 0.0 | -0.2 | -0.4 | -0.1 | 0.2 | 0.0 | -0.4 | 0.2 | 0.2 | 0.1 | -0.5 | 0.2 | 0.0 | 1.2 | 0.5 |
| NDUFS7 | -0.2 | | 0.4 | 0.2 | -0.7 | 0.0 | 0.2 | -0.1 | -0.5 | 0.4 | 0.2 | -0.2 | -1.0 | 0.7 | 0.2 | 1.3 | 0.6 |
| NDUFS8 | -0.2 | | 0.2 | -0.1 | -0.6 | 0.0 | 0.4 | 0.2 | 0.0 | 0.3 | 0.1 | 0.2 | -0.6 | 0.3 | 0.3 | 1.4 | 0.7 |
| NDUFV1 | -0.4 | | -0.2 | -0.1 | -0.9 | -0.1 | 0.0 | -0.3 | -0.8 | 0.1 | 0.2 | 0.3 | -1.0 | 0.1 | -0.2 | 1.1 | 0.2 |
| NDUFV3 | -0.2 | | 0.2 | -0.2 | -0.5 | 0.1 | 0.6 | 0.5 | -0.1 | 0.4 | 0.0 | 0.5 | -0.1 | 0.4 | 0.1 | 1.3 | 0.1 |
| UQCRFS1 | -0.4 | | -0.2 | -0.1 | -0.3 | -0.1 | 0.1 | -0.1 | -0.6 | -0.3 | 0.0 | 0.1 | -0.2 | 0.1 | -0.3 | 1.4 | 0.2 |
| UQCRH | -0.2 | | 0.2 | -0.2 | 0.3 | 0.0 | 0.1 | 0.6 | 0.0 | -0.3 | -0.1 | 0.0 | 0.3 | 0.0 | 0.1 | 1.1 | 0.4 |
| UQCRQ | -0.4 | | 0.1 | -0.2 | -0.3 | 0.0 | 0.2 | 0.2 | -0.1 | 0.4 | 0.4 | 0.6 | 0.2 | -0.1 | 0.0 | 1.4 | 0.5 |
| System lupus erythemous | | | |  |  |  |  |  |  |  |  |  |  |  |  |  |  |
| HIST2H2AA3 | | -0.6 | 0.1 | 0.1 | -1.3 | 0.1 | 0.2 | -0.2 | -1.0 | 0.9 | 0.3 | -0.5 | -1.8 | -0.7 | 0.1 | 0.6 | -1.3 |
| HIST1H2AA | | -0.4 | 0.0 | -0.1 | -0.2 | -0.6 | -0.6 | -1.5 | -1.7 | 0.3 | -0.4 | -0.4 | -0.3 | -0.1 | 0.0 | -0.1 | -0.2 |
| HIST1H4B | | -0.9 | 0.1 | 0.0 | -1.9 | 0.2 | -0.1 | 0.4 | -0.4 | 0.3 | 0.0 | 0.4 | -0.7 | -0.2 | 0.0 | 0.7 | -0.5 |
| HIST2H2AC | | -0.6 | 0.1 | 0.1 | -1.1 | 0.2 | 0.2 | 0.0 | -0.8 | 0.8 | 0.3 | -0.1 | -1.2 | -0.6 | 0.1 | 0.7 | -1.0 |
| HIST1H2AI | | -0.5 | 0.0 | 0.0 | -1.0 | 0.1 | 0.2 | -0.2 | -0.8 | 0.9 | 0.3 | 0.3 | -1.2 | -0.3 | 0.2 | 0.7 | -0.8 |
| HIST3H2A | | -0.5 | 0.0 | 0.0 | -1.0 | 0.1 | 0.2 | -0.2 | -0.8 | 0.9 | 0.3 | 0.3 | -1.2 | -0.3 | 0.2 | 0.7 | -0.8 |
| H2AFX | | -0.5 | 0.2 | 0.0 | -1.9 | 0.3 | 0.3 | 0.4 | -1.4 | 0.9 | 0.3 | 0.1 | -1.4 | -0.4 | 0.3 | 1.0 | -1.4 |
| H2AFI | | -0.4 | 0.2 | 0.1 | -1.8 | 0.4 | 0.6 | 0.4 | -1.4 | 1.0 | 0.4 | 0.2 | -1.5 | -0.3 | 0.2 | 1.0 | -1.3 |
| HIST1H3G | | -0.1 | -0.1 | 0.0 | -1.0 | 0.2 | 0.3 | 0.3 | -0.5 | 0.1 | 0.2 | -0.6 | -1.0 | -0.1 | 0.0 | 0.2 | -0.6 |
| Chemokine signaling pathway | | | | | | | | | | | | | | | | | |
| CXCL1 | -1.1 | | -1.7 | -1.1 | -1.4 | -0.2 | 0.0 | -0.3 | -0.4 | 0.0 | 0.0 | 0.2 | 0.0 | -0.2 | -0.1 | 0.3 | 0.2 |
| CCL2 | -0.6 | | -1.4 | -0.1 | 0.1 | -0.1 | -0.3 | 0.4 | 0.0 | -0.1 | -0.7 | -0.2 | 0.1 | -0.6 | 0.2 | -0.5 | 0.7 |
| CCR5 | 0.3 | | -0.1 | 0.2 | 0.1 | -0.2 | 0.1 | -0.1 | 0.0 | 0.0 | 0.0 | 0.4 | 0.2 | -1.2 | 0.4 | -0.9 | -0.1 |
| CXCL13 | 0.3 | | 0.0 | -0.2 | 0.1 | 0.2 | 0.0 | 0.1 | 0.1 | 0.3 | 0.2 | 0.0 | 0.1 | 2.3 | 0.0 | 0.3 | 0.9 |
| CCL19 | -0.2 | | -0.1 | 0.2 | 0.1 | 0.2 | -0.2 | 0.0 | 0.1 | 0.5 | 0.3 | 0.1 | 0.8 | 1.3 | 0.2 | 0.3 | 0.5 |
| CXCL12 | 0.3 | | 0.8 | 0.5 | 0.7 | 0.4 | 0.5 | -0.3 | -0.1 | 0.2 | 0.1 | 0.1 | 0.2 | 1.1 | 0.1 | -0.3 | 0.4 |

Data is shown as signal log2 ratio to those of control ZDF group. N, Normal. G, Glimepiride. R, Rosiglitazone. M, Metformin.
