# Supplementary material for: Transcriptomic Analysis of Insulin-Sensitive Tissues from Anti-Diabetic Drug Treated ZDF Rats, a T2DM Animal Model
Source: PLoS One. 2013 Jul 26;8(7):e69624. doi: 10.1371/journal.pone.0069624 (PMC3724940; doi:10.1371/journal.pone.0069624)
Supplement: Table S3 — Sequences of the rat real-time primer pairs used. (DOCX) [file pone.0069624.s004.docx]

| Gene Symbol | Accession # |  |  | Size |
| --- | --- | --- | --- | --- |
| Atp5d | NM_139106 | Forward | AGTCGTGACACTGGACATGC | 248 |
|  |  | Reverse | TAAGCCAGTAACCGGAGCAC |  |
| Cox5b | NM_053586 | Forward | TCCCTACTGATGAGGAGCAG | 169 |
|  |  | Reverse | CAGATGCAGCCCACTATTCT |  |
| Ndufa8 | NM_001047862 | Forward | CTGCTCTCTTTGGTGATGGT | 291 |
|  |  | Reverse | GTCAAACTTTGCCTGCTGTT |  |
| Ndufa9 | NM_001100752 | Forward | GGATTGCTAACGCGACTAAA | 215 |
|  |  | Reverse | CACCTTGTCCTTTGTTGTCC |  |
| Ndufab1 | NM_001106294 | Forward | ACAGTTTGGACCAAGTGGAA | 209 |
|  |  | Reverse | TGGTTCACTCTTGCTTGTCA |  |
| Ndufb2 | NM_001108624 | Forward | TTGGAGATTTTGGCATGACT | 208 |
|  |  | Reverse | TCCTCATTTTGGCAAGCTAC |  |
| Ndufb6 | NM_001106646 | Forward | CTGGAGCGATTCTGGAATAA | 191 |
|  |  | Reverse | GGAAATATTCTGGGCTTCGT |  |
| Ndufb8 | NM_001106360 | Forward | TTTGTGGCTTTCATGGTTTT | 204 |
|  |  | Reverse | AGGAACAAGGAGTCCCATTC |  |
| Ndufs7 | NM_001008525 | Forward | GGCACGCTCACTAACAAGAT | 174 |
|  |  | Reverse | TGGCACATAGATGTCCACTG |  |
| Ndufs8 | NM_001106322 | Forward | TAATGGCCAAAGCCTTCATA | 200 |
|  |  | Reverse | CAAAGGGGTAGTTGATGGTG |  |
| Ndufv1 | NM_001006972 | Forward | GCAAAAATGCCTGTGACTCT | 163 |
|  |  | Reverse | CCAAATACTCCCACATCTGC |  |
| Ndufv3 | NM_022607 | Forward | AGAGAGCTGGACCACAACTG | 160 |
|  |  | Reverse | TGATGCTGCAGGTTCTTGTA |  |
| Uqcrfs1 | NM_001008888 | Forward | TACTGTGGGTGTTGCATACG | 230 |
|  |  | Reverse | GGTCCCTTAACTGGGACACT |  |
| Uqrch | NM_001009480 | Forward | TGGGACTAGAGGATGAACGA | 181 |
|  |  | Reverse | GTCTGTGATCGGGAAGACAC |  |

**Table S3. Sequences of rat real-time primer pairs used**
