# Supplementary material for: Transcriptomic Analysis of Insulin-Sensitive Tissues from Anti-Diabetic Drug Treated ZDF Rats, a T2DM Animal Model
Source: PLoS One. 2013 Jul 26;8(7):e69624. doi: 10.1371/journal.pone.0069624 (PMC3724940; doi:10.1371/journal.pone.0069624)
Supplement: Table S5 — GSA with the DEGs commonly regulated by metformin treatment. (DOCX) [file pone.0069624.s006.docx]

**Table S5. GSA with commonly regulated DEG by metformin treatment.**

| Pathway | Count | PValue | Genes |
| --- | --- | --- | --- |
| *Up-regulted* |  |  |  |
| rno03010:Ribosome | 32 | <10^-3^ | LOC680170, LOC365569, LOC685887, RPL39, LOC501769, LOC364390, LOC691434, LOC298509, LOC367275, LOC690022, RPL30, LOC691975, LOC680393, RPL32, LOC681330, LOC287947, RPL4, RPS21, LOC364402, LOC296165, LOC365655, RGD1563570, RGD1561618, RGD1562796, LOC680395, LOC501835, LOC680988, LOC367398, RPL22, LOC366632, LOC367943, LOC366928 |
| rno03320:PPAR signaling pathway | 7 | <10^-3^ | ACOX1, EHHADH, FABP7, ACSL3, CPT1A, NR1H3, ACSL5 |
| rno00100:Steroid biosynthesis | 4 | 0.002 | TM7SF2, EBP, SC5DL, FDFT1 |
| rno00071:Fatty acid metabolism | 5 | 0.004 | ACOX1, EHHADH, ACSL3, CPT1A, ACSL5 |
| *Down-regulated* |  |  |  |
| rno04512:ECM-receptor interaction | 10 | <10^-3^ | COL4A1, LAMC3, ITGA7, COL3A1, COL1A2, DAG1, COL6A2, COL2A1, COL1A1, COL5A1 |
| rno04510:Focal adhesion | 15 | <10^-3^ | COL4A1, VAV3, BRAF, COL3A1, MYLK2, PRKCG, COL2A1, COL5A1, MYL9, LAMC3, JUN, ITGA7, COL1A2, COL6A2, COL1A1 |
| rno04810:Regulation of actin cytoskeleton | 15 | <10^-3^ | GIT1, ARHGEF1, VAV3, BRAF, ARHGEF7, BAIAP2, SSH3, MYLK2, ARPC5, MYH9, MYL9, MK1, ITGA7, LOC685513, MYH14 |
| rno05416:Viral myocarditis | 8 | 0.004 | EIF4G1, RT1-A1, DAG1, LOC683761, MYH7, MYH14, MYH9, RT1-N1 |
| rno04144:Endocytosis | 11 | 0.018 | GIT1, RT1-A1, FAM125A, ARRB2, RAB5C, CHMP4B, RAB4A, AP2S1, LOC683761, HSPA1A, RT1-N1 |
| rno04530:Tight junction | 8 | 0.032 | MAGI1, EPB4.1, MYH7, PRKCG, MYH14, MYH9, LLGL2, MYL9 |
| rno04010:MAPK signaling pathway | 12 | 0.046 | ARRB2, DUSP1, BRAF, RPS6KA2, JUN, GADD45G, LOC685513, HSPB1, PRKCG, HSPA1A, IKBKB, GADD45A |
| rno04270:Vascular smooth muscle contraction | 7 | 0.048 | EDNRA, ARHGEF1, BRAF, MYLK2, PRKCG, ARHGEF11, MYL9 |
